# Cognitive reserve is associated with the functional organization of the brain in healthy aging: a MEG study

**DOI:** 10.3389/fnagi.2014.00125

**Published:** 2014-06-13

**Authors:** María E. López, Sara Aurtenetxe, Ernesto Pereda, Pablo Cuesta, Nazareth P. Castellanos, Ricardo Bruña, Guiomar Niso, Fernando Maestú, Ricardo Bajo

**Affiliations:** ^1^Laboratory of Cognitive and Computational Neuroscience (UCM-UPM), Centre for Biomedical Technology, Universidad Politécnica de MadridMadrid, Spain; ^2^Department of Basic Psychology II, Complutense University of MadridSpain; ^3^Grupo de Ingeniería Eléctrica y Bioingeniería, Department of Industrial Engineering and Institute of Biomedical Technology, Universidad de La LagunaLa Laguna, Tenerife; ^4^Departamento de Matemáticas, Universidad Internacional de La Rioja (UNIR)Logroño, Spain

**Keywords:** brain efficiency, cognitive reserve, functional connectivity, healthy aging, MEG

## Abstract

The proportion of elderly people in the population has increased rapidly in the last century and consequently “healthy aging” is expected to become a critical area of research in neuroscience. Evidence reveals how healthy aging depends on three main behavioral factors: social lifestyle, cognitive activity, and physical activity. In this study, we focused on the role of cognitive activity, concentrating specifically on educational and occupational attainment factors, which were considered two of the main pillars of cognitive reserve (CR). Twenty-one subjects with similar rates of social lifestyle, physical and cognitive activity were selected from a sample of 55 healthy adults. These subjects were divided into two groups according to their level of CR; one group comprised subjects with high CR (9 members) and the other one contained those with low CR (12 members). To evaluate the cortical brain connectivity network, all participants were recorded by Magnetoencephalography (MEG) while they performed a memory task (modified version of the Sternberg's Task). We then applied two algorithms [Phase Locking Value (PLV) and Phase Lag Index (PLI)] to study the dynamics of functional connectivity. In response to the same task, the subjects with lower CR presented higher functional connectivity than those with higher CR. These results may indicate that participants with low CR needed a greater “effort” than those with high CR to achieve the same level of cognitive performance. Therefore, we conclude that CR contributes to the modulation of the functional connectivity patterns of the aging brain.

## Introduction

Over the last decade, there has been increasing interest in the study of the cognitive decline associated with normal aging. In fact, demographic studies provide evidence of the progressive inversion of the population pyramid, such that it is expected that by the year 2100, 22.3% of people will be aged 65 or over, as compared to a mere 7.6% in 2010 (source: United Nations). Thus, understanding aging is rightly considered a scientific and social priority warranting detailed investigation of the basic mechanisms associated with healthy aging.

It is well-known that cognitive capacity diminishes with aging (Fergus and Timothy, 1992; Grady, 2012). Evidence reveals that healthy aging is influenced by protective factors such as social lifestyle, cognitive and physical activity. In their review, Fratiglioni et al. (2004) claimed that “an active and socially integrated lifestyle in late life seems to protect against Alzheimer's disease and dementia.”

Encompassed within cognitive activities, educational level and occupational attainment through life have been the two most studied factors and considered the strongest predictors of the maintenance of cognitive abilities in the aging process (see e.g., Stern et al., 1994; Anstey and Christensen, 2000; Ardila, 2000). These variables are involved in the concept of cognitive reserve (CR), which refers to the ability to tolerate age-related changes and disease-related pathologies in the brain, without developing clear clinical symptoms (Stern et al., 2012).

From a neurophysiological point of view, two non-mutually exclusive models have been proposed for the underlying reserve mechanism (Stern, 2002). At an anatomical level, the passive or brain reserve capacity (BRC) model considers direct measures of brain, such as brain volume or the number of synapses, to be factors underlying reserve, and assumes that larger brains present higher endurance capacity to neuropathology (Katzman et al., 1988; Mortimer et al., 2003). At a functional level, the active or CR's model considers the ability to recruit brain networks in an effective way to be the factor underlying reserve (Stern, 2002; Habeck et al., 2003; Scarmeas et al., 2003, 2004; Stern et al., 2005). According to this model, once pathological processes or cognitive decline begin to occur, CR uses the existing brain networks more efficiently to successfully perform a particular task (neural reserve) or alternative networks to maintain a normal cognitive status (neural compensation) (Stern et al., 2012). It is important to emphasize that although initially proposed as independent processes, brain reserve and CR concepts are not mutually exclusive and are both involved in providing protection against brain damage (Stern, 2006).

Several studies have tried to determine the anatomical and functional mechanisms/features underlying CR. These studies have provided evidence that CR contributes to modulate brain activity and effectiveness when executing cognitive tasks (Habeck et al., 2003; Scarmeas et al., 2003; Bartrés-Faz et al., 2009; Solé-Padullés et al., 2009; Stern et al., 2012). Results from these studies reveal that, in order to achieve a similar level of performance during cognitive tasks, participants with low CR show higher brain activation than participants with high CR. However, to our knowledge, no research has studied the functional architecture of the networks involved in these processes.

Nowadays, one of the main approaches used for this purpose is the concept of functional connectivity. This term has been coined to describe how brain regions are coordinated to support higher cognitive functions (Friston, 1994, 2001; Sporns et al., 2004). Functional connectivity reflects the statistical dependencies between two physiological signals, which provide relevant information about functional interactions between the corresponding brain regions. Long-range synchronization between the oscillatory activity of brain signals originating in relatively distant neuronal populations has been proposed as the mechanism for communication and integration of information in the brain (Varela et al., 2001; Fries, 2005), and it is also present during spontaneous oscillatory activity (Hipp et al., 2012). Therefore, the characterization of functional connectivity is a suitable approach to study brain functioning (Singer, 1999; Buzsáki and Draguhn, 2004), and hence the CR.

Magnetoencephalography (MEG) facilitates such an approach, with its signals providing a direct measure of neuronal activity, which allows the dynamics of the cortical networks underlying cognition to be studied. By applying functional connectivity indexes to MEG data, it is possible to infer the topology and efficiency of functional neural networks underlying cognitive processes (De Haan et al., 2012; Siegel et al., 2012). However, as far as we know, no research has used functional connectivity to analyze MEG signals underlying CR during the execution of a cognitive task.

Thus, this work aimed to study the relationship between CR, brain dynamics and cognitive performance in healthy aging. As educational and occupational attainment throughout life have been proposed as adequate proxies for brain and CR models (Stern, 2002, 2009), we chose these two variables to characterize participants in this study. Therefore, participants were first divided into two groups according to their score on a CR index (CRI) proposed by Garibotto et al. (2012), which was based on both proxy measures of reserve (see Materials and Methods for the explanation). To ensure adequate isolation of these two variables, we controlled the other factors which may be also involved in CR, such as social, physical, and cognitive activities realized at present. Then, MEG signals were recorded during the execution of a cognitive task. Specifically, we chose a memory task (Sternberg's modified Task) because cognitive capacity diminishes with aging, with memory decline being one of its main features (Grady, 2012). We then used two phase synchronization (PS) indexes [Phase Locking Value (PLV) and Phase Lag Index (PLI)], which assess the degree of functional connectivity between oscillatory narrow-band signals, in order to characterize the functional topology of the neural network of each group while performing the memory task.

Based on the existing literature, we hypothesized that participants with lower educational and occupational attainment would reach a similar level of performance but showing higher connectivity values in most of the frequency bands analyzed, as compared to participants with higher educational and occupational attainment.

## Materials and methods

### Participants

Twenty-one healthy adults enrolled in the study. All of them were recruited from the “Geriatric Unit of the University Hospital San Carlos” (Madrid, Spain). All subjects fulfilled the following inclusion criteria: (1) right handed (Oldfield, 1971); (2) native Spanish speakers; (3) between 65 and 85 years old; (4) a Mini-Mental State Examination (MMSE, Lobo et al., 1979) score greater than 28; (5) no memory impairment documented by delayed recall from the Logical Memory II subtest of the Wechsler Memory Scale Revised (WMS-III-R; Wechsler, 1987), (cut-off scores >16 for participants with more than 15 years of education; >8 for participants with 8–15 years of education); and (6) normal daily living activities (assessed with the Spanish version of the Functional Assessment Scale Pfeffer et al., 1982). Exclusion criteria included: (1) medical history of psychiatric or any other neurological disease; (2) psychoactive drugs consumption; and (3) severe sensory or comprehension deficits.

**Figure 1:**
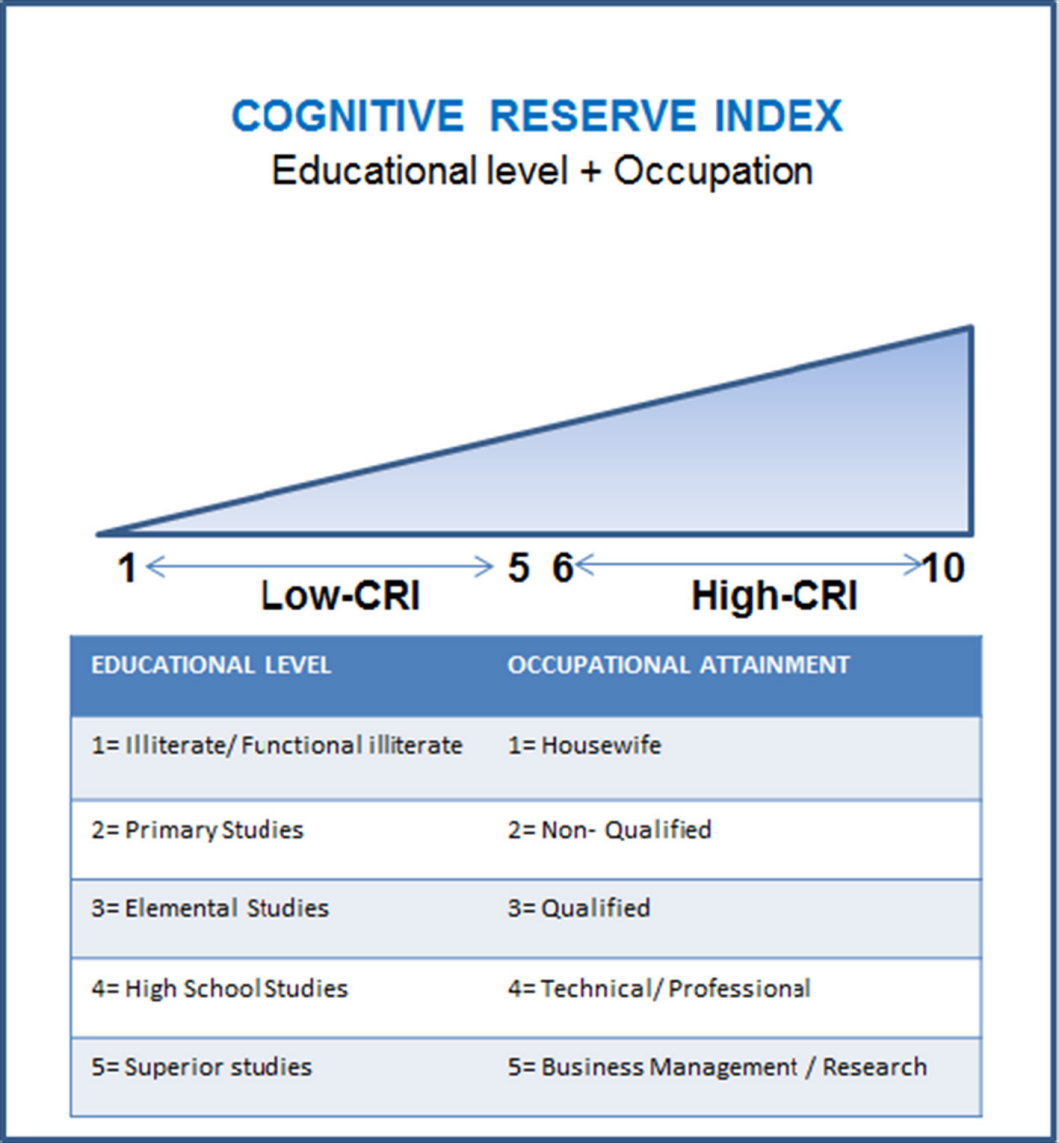
**Cognitive reserve index (CRI) calculation**. Educational level was grouped into five levels: (1) illiterate/functional illiterate, (2) primary studies, (3) elemental studies, (4) high school studies, (5) superior studies. Occupational attainment was divided into five levels: (1) housewife, (2) non-qualified, (3) qualified; (4) technical/professional; (5) business management/research.

We considered the educational level and the occupational attainment to classify the subjects according to a scale (see Figure 1). Education was measured depending on the formal education attained and occupational attainment taking into account the main professional activity carried out during the active life of each participant. Moreover, current physical, cognitive, and social activities performed by each participant were subsequently assessed by a self-reported questionnaire, where the score was based on the weekly frequency of each activity (see Table 1). In the physical category, we included the following activities: swimming, running, walking, or going to the gym; in the cognitive category these others: knitting, listening to music or to the radio, reading (books, magazines, newspapers), playing games (cards, parcheesi), going to classes, to a club or center, watching TV, going to the cinema, theater, or sport events, participating in cultural activities or other hobbies. Finally, in the social category we considered: visiting friends or family and participating in social activities.

**Table 1:** **Description of the scores of cognitive, physical, and social activities per week**.

**Activities**	**Hours per week**							
Cognitive	0 (0 points)	1–3 (1 point)	4–7 (2 points)	8–10 (3 points)	11–14 (4 points)	15–17 (5 points)	18–20 (6 points)	>21 (7 points)
Physical	0 (0 points)	1–2 (1 point)	3–5 (2 points)	6–7 (3 points)	8–10 (4 points)	11–13 (4 points)	>14 (6 points)	
Social	0 (0 points)	1–2 (1 point)	3–4 (2 points)	>5 (3 points)				

In order to study the effect of education and occupational attainment (CRI factors) during the execution of a cognitive task, the initial 55 participants were matched for physical, cognitive, and social activities during late-life. Therefore, 21 participants were finally included in the study. Then, they were divided in two different subgroups according to the CRI, which was calculated by adding two independent categories: the educational level (maximum score of 5) and the occupational attainment (maximum score of 5) (Garibotto et al., 2012), Table 2 summarizes the features of each group. Based on this CRI, we considered that a subject with scores between 1 and 5 belonged to the low CRI group, whereas those with scores between 6 and 10 were classified into the high CRI group (see Figure 1). With this procedure, the low CRI group was finally formed by 12 subjects, whereas the high CRI group was formed by 9 subjects (see Table 2).

**Table 2:** **Description of the mean and standard deviations of each group**.

	**High-CRI**	**Low-CRI**	**Sig**.
Age	67.3 ± 7.4	69.7 ± 6.6	*p* > 0.05
Educ. level	4.5 ± 0.7	2.4 ± 0.7	*p* < 0.05^*^
Occupation	4.5 ± 0.5	1.6 ± 0.5	*p* < 0.05^*^
CRI	9 ± 0.6	3.7 ± 1.1	*p* < 0.05^*^
Cognitive act.	4.2 ± 2.3	2.8 ± 1.4	*p* > 0.05
Physical act.	2.4 ± 1.5	2.7 ± 1	*p* > 0.05
Social act.	1.6 ± 1.2	1.7 ± 1	*p* > 0.05
Acc. Smaqe	114 ± 10.7	106 ± 14.1	*p* > 0.05

* Statistically significant values are p < 0.05.

Statistically significant values are p < 0.05^*^. Groups (High and Low CRI) differ in educational level and occupation and therefore, in CR, but they are not different otherwise. Accuracy of the memory task performance (Acc. Smaqe) did not differ significantly between groups.

Before MEG recordings, all participants gave written informed consent to participate in the study, which was approved by the Local Ethics Committee.

### Stimuli and task

**Figure 2:**
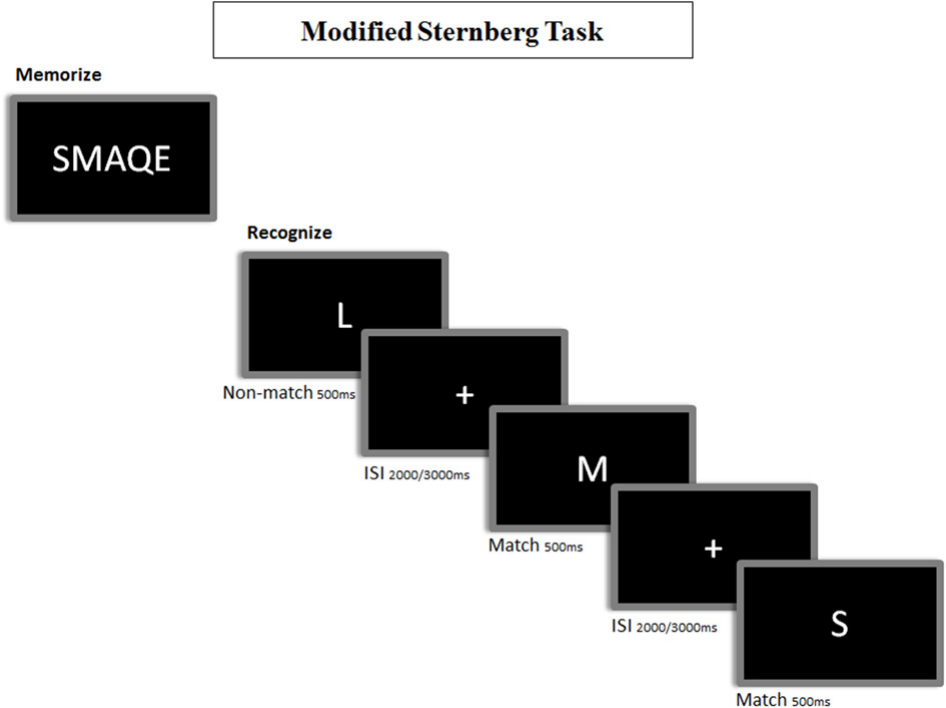
**Representation of the memory task paradigm**. In the encoding phase, participants are instructed to memorize 5 letters (i.e.: “SMAQE”). In the recognition phase, participants are instructed to make a match/non-match button-press to indicate that the presented letter matched any of the encoded ones.

A modified version of the Sternberg's letter probe task (deToledo-Morrell et al., 1991) was used (see Figure 2). A set of five letters was presented to the participants, who were then asked to keep them in mind. After the presentation of the five letter set, a series of single letters (500 ms in duration, with a random ISI between 2 and 3 s) was presented one at a time, and the participants were asked to press a button with their right hand when a member of the initial set was detected. The list consisted of 250 letters, in which half were targets (i.e., they were present in the initial set), and half distracters (i.e., they were not). All participants completed a training session before the actual test, which did not start until each subject demonstrated that he/she could remember the five letter set. Letters were projected through a LCD video projector (SONY VPLX600E), situated outside of the shielded room onto a series of in-room mirrors, the last of which was suspended approximately 1 meter above the participant's face. The letters subtended 1.8 and 3 degrees of horizontal and vertical visual angle, respectively.

### MEG data collection

MEG signals were recorded during the execution of the modified Sternberg's task described above, with a 254 Hz sampling frequency and a band pass of 0.5 to 50 Hz, using a 148 channel whole-head magnetometer (MAGNES 2500 WH, 4D Neuroimaging) confined in a magnetically shielded room. An environmental noise reduction algorithm using reference channels at a distance from the MEG sensors was applied to the data. Thereafter, single trial epochs were visually inspected by an experienced investigator, and epochs containing visible blinks, eye movements, or muscular artifacts were excluded from further analysis. Artifact free epochs from each channel were then classified into four different categories, according to the subject's performance in the experiment: hits, false alarms, correct rejections, and omissions. Only hits were considered for further analysis, because we were interested in evaluating the functional connectivity patterns that support recognition success. Thirty-five epochs per subject were used to calculate PLV and PLI values. This lower bound was determined by the participant with least epochs. So, to have an equal number of epochs across participants, the 35 epochs were randomly chosen from each of the other participants.

### MEG data analysis

#### PLV and PLI

All epochs were band-pass filtered off-line in frequency bands of 4 Hz in the range between 4 and 44 Hz: 4–8 Hz, 8–12 Hz, 12–16 Hz,…, 40–44 Hz. Subsequently, PLV and PLI values were calculated for each of the 35 1-s epochs to estimate the degree of PS within each of these frequency bands for each of the 148^*^147/2 sensors pairs using the HERMES toolbox (Niso et al., 2013). PLV makes use of the relative phase difference (Lachaux et al., 1999; Mormann et al., 2000). It is defined as:

(1) $$\begin{array}{cc} \begin{array}{l} PLV=\;|\;\langle e^{i\Delta \phi \left(t_{n}\right)}\rangle |\;=\;|\;\frac{1}{N}\sum_{n\;=\;1}^{N}e^{i\Delta \phi \left(t_{n}\right)}|\\ \;=\sqrt{{\langle \cos\Delta \phi \left(t\right)\rangle }^{2}+{\langle \sin\Delta \phi \left(t\right)\rangle }^{2}} \end{array} & \left(1\right) \end{array}$$

where <.> indicates time average and Δϕ *(t)* is the cyclic relative phase at time *t*, i.e., the difference between the phase of signals x(t) and y(t) wrapped to the interval [0,2π)

The PLV estimates how the relative phase is distributed over the unit circle. When there is strong PS between x(t) and y(t), Δϕ *(t)* occupies a small portion of the circle and the PLV is close to 1, whereas if the signals are not synchronized, the relative phase would spread out all over the unit circle and the PLV would remain low. Note that PLV is not robust against the presence of common sources (e.g., volume conduction effects).

Instead, the PLI (Stam et al., 2007), discards phase differences that centered around 0 mod π in order to be robust against the presence of common sources (volume conduction).

(2) $$PLV=\left|\;\langle sign\left(\Delta \phi \left(t_{n}\right)\right)\rangle \right|\;=\;|\frac{1}{N}\sum_{n\;=\;1}^{N}sign\left(\Delta \phi \left(t_{n}\right)\right)|$$

The range of this index is 0 ≤ *PLI* ≤ 1. (0): no coupling or coupling with a phase difference centered around 0 or π, (1): perfect phase locking at a value of Δϕ *(t)* different from 0 mod π.

### Statistical analysis

Once PLV and PLI values were calculated, a between-group Mann-Whitney *U*-test (non-parametric test) was performed for each pair of sensors and the two groups of subjects [high CR (9 members) and low CR (12 members)]. Thus, we obtained a symmetric squared matrix (148 × 148) containing all the corresponding p values.

Subsequently, to avoid the multiple comparisons problem, we performed a non-parametric permutation testing (Ernst, 2004). This was done by randomly dividing the participants into two sets, matching the numbers of the original groups (9 and 12). Besides, for each subject, PLV and PLI values were randomized.

Then, a Mann-Whitney *U*-test between each pair of sensors was then carried out between these two newly created groups. The procedure was repeated 2000 times and the *p*-value from each test for sensor pair was calculated to obtain a *p*-value distribution for each channel pair. We then identified the 1th percentile of each distribution, and only those *p*-values below that threshold were considered significant.

## Results

### Demographic results

High- and low-CRI participants were matched in age [*t*_(20)_ = 0.8, *p* = 0.4], cognitive [*t*_(20)_ = 1.6, *p* = 0.1], physical [*t*_(20)_ = 0.6, *p* = 0.5], and social [*t*_(20)_ = 0.1, *p* = 0.9] activities. Analysis of the task performance revealed no differences between groups [*t*_(20)_ = 1.4, *p* = 0.1].

### Connectivity results

**Figure 3:**
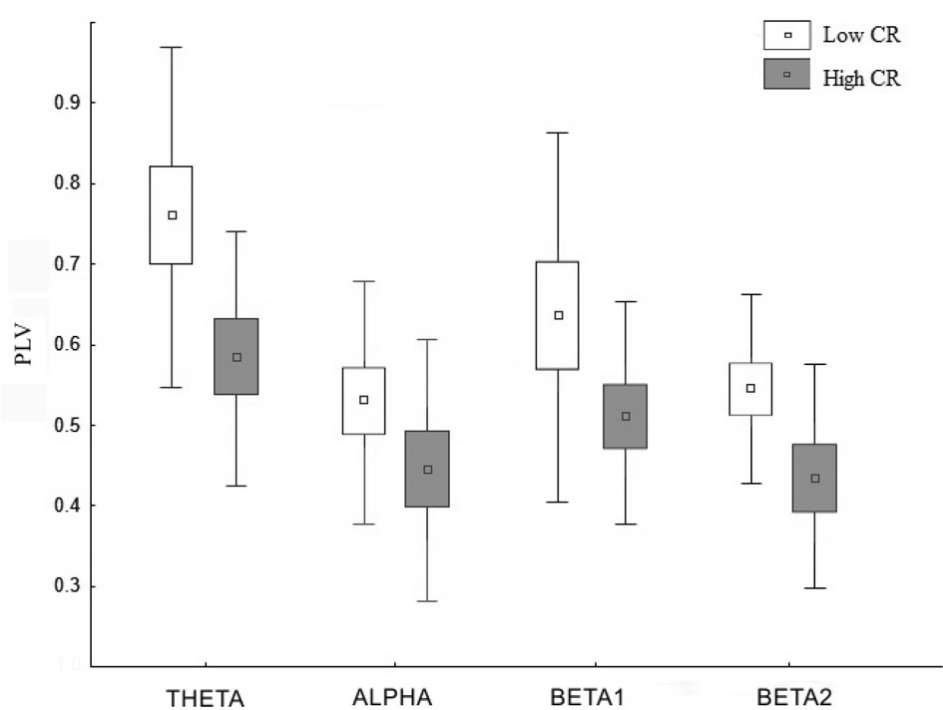
**Distribution of the PLV for the frequency bands theta, alpha, beta 1, and beta 2 in those regions showing significant differences between the low cognitive (blank) and the high cognitive reserve group (gray)**. The squares, boxes, and whiskers stand for the medians the 75% and the 99% quartiles of the distribution, respectively.

**Figure 4:**
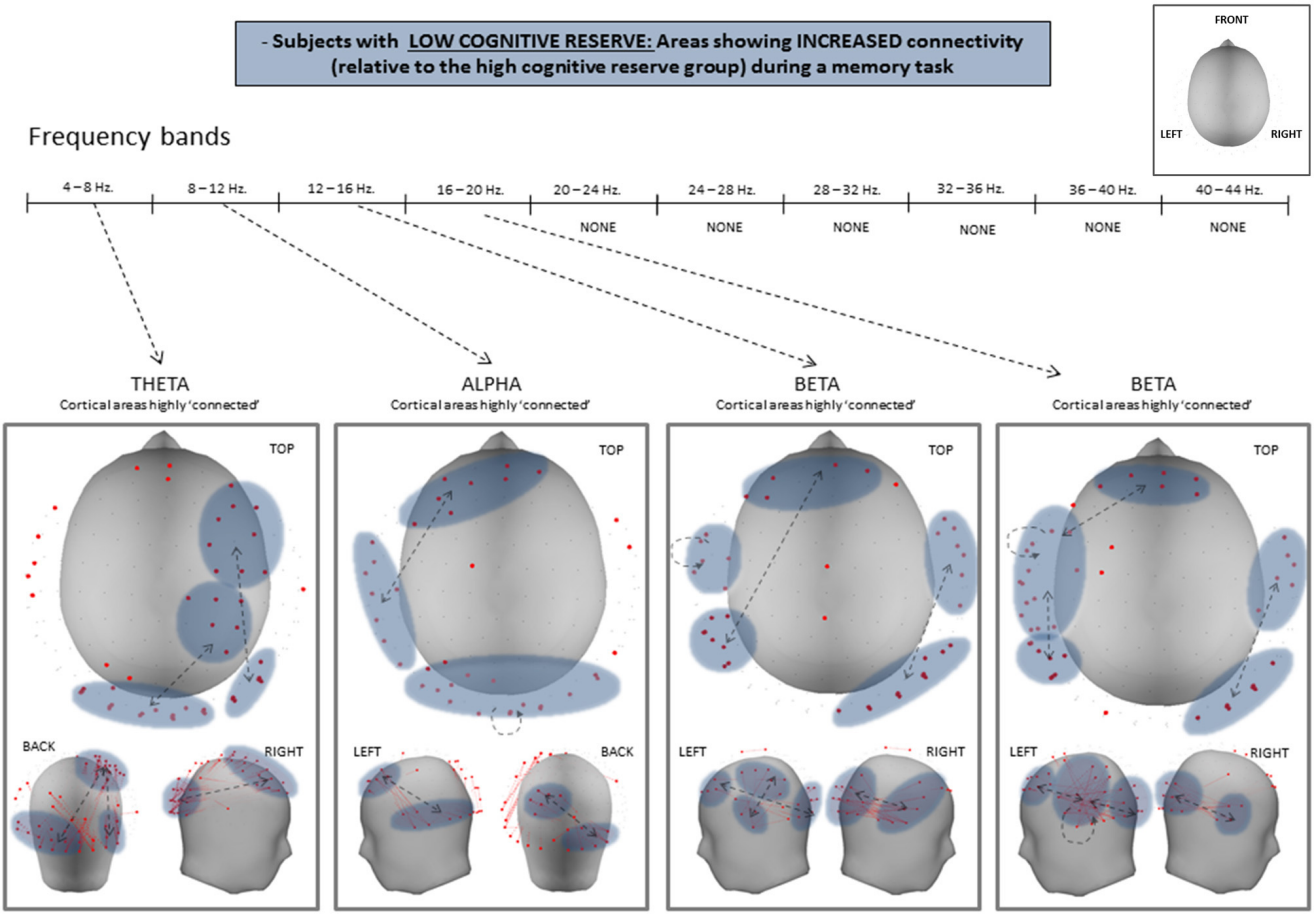
**Statistical differences of PLV between high- and low-CRI subjects in theta, alpha, beta 1, and beta 2 frequency bands**.

Statistical analysis of both PLV and PLI values revealed an overall increase in connectivity in the low CRI participants, compared to the high CRI subjects in the following frequency bands: 4–8 Hz (theta), 8–12 Hz (alpha), 12–16 Hz (beta 1), and 16–20 Hz (beta 2) (see Figure 3 and Table 3), but located in different sensors (see Figure 4). Thus, differences in synchronization in theta band between both groups were observed between right fronto-occipital sensors and between right parietal-occipital sensors. In alpha band low CRI participants showed higher synchronization values between fronto-temporal sensors of the left hemisphere and within occipital sensors. Differences in beta 1 band were observed between fronto-left parietal sensors and right temporo-occipital sensors and within left temporal sensors. Finally, differences in beta 2 band were observed between fronto-left temporal sensors, between left temporo-parietal sensors, between right temporo-occipital areas y and within left temporal sensors (Figure 4).

**Table 3:** Percentage over total number of links (pairs of sensors) (Nlinks = 148 * 147/2 = 10870), showing significant differences between both groups for the frequency bands studied (theta, alpha, beta 1, and beta 2).

**Frequency bands**	**Percentage over total number of links (Nlinks): Low CR > high CR**	**Percentage over total number of links (Nlinks): Low CR < high CR**
Theta	~1% (1027 links statistically significant)	~0% (4 links statistically significant)
Alpha	~0.6% (604 links statistically significant)	~0% (7 links statistically significant)
Beta 1	~0.8% (878 links statistically significant)	~0% (45 links statistically significant)
Beta 2	~0.7% (757 links statistically significant)	~0% (27 links statistically significant)

## Discussion

This study explored the relation between brain activity architecture and educational and occupational attainment throughout life (as assessed by an index of CR, the CRI), during the performance of a memory task in healthy aging.

For this purpose, healthy elderly subjects were grouped according to their different CR profiles, which were based on the two principal factors of CR. Thus, we studied two different groups based on the CRI: low and high CR, having controlled for age, physical, cognitive, and social current activities; in order to specifically assess the influence of CR on the organization of the functional networks. Then, we applied functional connectivity analysis to MEG time series by using two PS indexes, which are a robust measure of the extent of information integration in the brain (Stam and van Straaten, 2012). Since it is generally accepted that the process of normal aging impairs synaptic function and neuronal communication (Morrison and Baxter, 2012), we proposed that these methods would provide new insights about how functional neural networks are shaped by CR in elderly.

Increased functional connectivity may be related to network efficiency (Buldú et al., 2011). In an elegant study Palva et al. (2010) proved that during performance of a working memory task, synchronization increased with memory load in young subjects. That is, the more difficult the task, the more tightly connected the brain network has to be to perform successfully. Similarly, (Zarahn et al., 2007) compared brain activity in young and elderly subjects during a Sternberg task that progressively increased in terms of memory load. They found that elderly subjects needed higher activation of their neuronal networks than the young subjects, due to lower efficiency of the elderly subjects' neuronal networks. These findings appeared to substantiate the fact that, to successfully perform the same task, subjects with lower educational and occupational attainment present increased brain connectivity as compared to subjects with higher level in these two variables. These results lead us to claim that, since individuals with a lower CRI need increased connectivity strength to perform the same cognitive task, their cortical networks are less efficient. That is, they need to overuse the brain functional networks to achieve a similar performance on the memory task, which would imply a decrease in network efficiency.

In summary, efficiency refers to the change in neural activity occurring with a change in task demand. Thus, greater efficiency would be associated with the recruitment of less neural activity and therefore less energy loss. As a consequence, under similar task performance, the more efficient network would be the one presenting lower brain synchronization and energy loss (for similar interpretations see Stern et al., 2005, 2012; Steffener and Stern, 2012). The discussion on connectivity related to efficiency is a relatively novel issue and has not yet been clarified. Bajo et al. (2010) showed that inter-hemispheric synchronization values increase reflected a compensatory mechanism for the lack of memory network efficiency in patients suffering from mild cognitive impairment (MCI). A network analysis revealed a non-efficient organization (Buldú et al., 2011) indicating that the increased functional connectivity it is not always associated with a better network functioning.

Modulation of brain activity during cognitive performance has also been found when decomposing EEG/MEG signals into spectral frequency bands. An increase in theta band activity has been observed during memory performance (Klimesch, 1996; Kim et al., 2012), especially in medial prefrontal regions (Raghavachari et al., 2001; Langer et al., 2013). Furthermore, coupling of theta band activity between several brain regions has been correlated with improving task execution (Axmacher et al., 2010; Fell and Axmacher, 2011; Langer et al., 2013). In a similar manner, alpha band activity has been related to active cognitive processes during memory tasks (Palva and Palva, 2007) especially between prefrontal and parietal regions (Grimault et al., 2009). Furthermore, an increase in alpha band activity during the detection of previously presented items suggests that this band plays a role in recognition processes (Van Strien et al., 2005). The activity in theta and alpha band has been associated with the amount of information to be retained (Klimesch, 1999; Jensen and Tesche, 2002; Jensen et al., 2002; Palva et al., 2010; Meeuwissen et al., 2011; Palva and Palva, 2011). Recently, Geerligs et al. (2014) compared young and elderly people and showed that an increase in connectivity in both alpha and beta bands was related to a better performance in older participants only, suggesting that this is an active compensation mechanism employed to maintain adequate performance. It seems likely that this normal increase in connectivity that is used as a compensatory mechanism in the elderly is being overused by low CRI subjects. Thus, these three frequency bands are related to the memory process and seem to be over-active in the CRI group, indicating a non-efficient organization of the functional networks.

Our findings agree with the aforementioned literature, inasmuch as they revealed that subjects with lower CRI present greater connectivity than those with higher CRI in the alpha and beta bands in the left hemisphere and in the theta band of the right hemisphere. This increased connectivity was observed in both anterior (prefrontal) and posterior (temporal, parietal, and occipital) regions. The present results regarding a high connectivity in low CRI participants over the left hemisphere accounts for the verbal nature of the memory task utilized. Given that all of our subjects were right-handed, the recruitment of the dominant hemisphere for the execution of the task reflects its role in mediating verbal memory processes. In the case of the right hemisphere, the recruitment of brain regions here seems to indicate compensatory mechanisms that contribute to the performance of the task. Brain bilateral activity in response to verbal information has been found in studies comparing young and elderly subjects and interpreted as a compensatory mechanism in the context of the HAROLD model (Cabeza et al., 2002; Reuter-Lorenz, 2002). Moreover, the over-recruitment of brain regions in elderly subjects has been observed when achieving the same accuracy as younger subjects (Morcom, 2003). Considering this context, and in agreement with our initial hypothesis, elderly individuals with lower CRI need to over-recruit more cortical networks, showing a lesser efficient brain functioning, to achieve the same level of cognitive performance as elderly individuals with higher CRI.

From a methodological point of view, the fact that our results are consistent with the two PS indexes used (similar results were obtained for both indexes, then only PLV outcomes were showed, Figure 4), which measure the existence of different types of coherent phase relationship between two signals, further supports the validity of these outcomes. In fact, the PLV does not distinguish between zero (i.e., 0 or π relative phase) and non-zero lag interdependence between two signals, the former of these phase relationships being normally ascribed to a mixture of one or more common neural source in the two sensors analyzed (Nolte et al., 2004). In contrast, PLI is nonzero if and only if this phase relationship is asymmetric, i.e., there is a certain time lag between the data, which is regarded as a sign of true direct connectivity (Nolte et al., 2004; Stam et al., 2007). However, zero-lag synchronization between two distant systems might occur if they are indirectly connected through a common relay (Fischer et al., 2006). This type of synchronization would result in a zero PLI between the two connected systems, which would not be the result of a common source (i.e., the same neural activity of a single brain area) measured on both of them. Yet such an indirect connection between cortical areas (which could be only detected using the PLV) with some deep brain structure acting as a dynamic relay, cannot certainly be ruled out (Gollo et al., 2011). This connectivity would indeed have a neurological origin rather than being a volume conduction artifact. Thus, the combined use of both indexes provides complementary, rather than confounding, information and our results suggest that both mechanisms (direct connectivity as assessed by the PLI and indirect connectivity / increased common source activity as assessed by the PLV) should be taken into account for measuring the brain efficiency during the execution of a cognitive task in subjects with different CR level.

One main limitation when interpreting the present results considers the size of the sample. For this reason, future studies should include a larger number of participants. Additionally, exploration of how cognitive demands shape the functional networks of the brain will contribute to characterize how functional neural networks are influenced by CR.

The current data shows the importance of quantifying the relation between the CR and the functional connectivity structure of the aging brain, and to evaluate its role in preventive and therapeutic strategies to achieve the goal of healthy aging.

### Conflict of interest statement

The authors declare that the research was conducted in the absence of any commercial or financial relationships that could be construed as a potential conflict of interest.
